# Computational Analysis of Mouse piRNA Sequence and Biogenesis

**DOI:** 10.1371/journal.pcbi.0030222

**Published:** 2007-11-09

**Authors:** Doron Betel, Robert Sheridan, Debora S Marks, Chris Sander

**Affiliations:** 1 Computational and Systems Biology Center, Memorial Sloan-Kettering Cancer Center, New York, New York, United States of America; 2 Department of Systems Biology, Harvard Medical School, Boston, Massachusetts, United States of America; Seoul National University, Republic of Korea

## Abstract

The recent discovery of a new class of 30-nucleotide long RNAs in mammalian testes, called PIWI-interacting RNA (piRNA), with similarities to microRNAs and repeat-associated small interfering RNAs (rasiRNAs), has raised puzzling questions regarding their biogenesis and function. We report a comparative analysis of currently available piRNA sequence data from the pachytene stage of mouse spermatogenesis that sheds light on their sequence diversity and mechanism of biogenesis. We conclude that (i) there are at least four times as many piRNAs in mouse testes than currently known; (ii) piRNAs, which originate from long precursor transcripts, are generated by quasi-random enzymatic processing that is guided by a weak sequence signature at the piRNA 5′ends resulting in a large number of distinct sequences; and (iii) many of the piRNA clusters contain inverted repeats segments capable of forming double-strand RNA fold-back segments that may initiate piRNA processing analogous to transposon silencing.

## Author Summary

The discovery of a new class of mammalian small regulatory RNAs termed PIWI-interacting RNA (piRNA) has extended the diverse family of small regulatory RNAs. PIWI proteins are a subclass of the larger Argonaute proteins family, of which the Ago members bind microRNAs and play a critical role in gene silencing. Despite the homology between PIWI and Ago proteins, piRNAs are strikingly different from microRNAs in their length, expression pattern, and genomic organization. In contrast, piRNAs are similar to repeat-associated small interfering RNA (rasiRNAs), a class of small RNAs that are responsible for transposon silencing in *Drosophila* germline, although it is unclear if piRNAs function in a similar way. This paper describes a computational comparison and analysis of the existing comprehensive piRNA datasets identified independently by three groups at the pachytene stage in mouse spermatogenesis. We find that the studies have identified similar genomic piRNA clusters, but differ substantially in the piRNAs that were cloned from those clusters. Based on these results we quantify the expected number of piRNAs and suggest that the processing of piRNAs from genomic transcripts is quasi-random. We find that a weak sequence signature may guide the piRNA 5′end processing that accounts for the departure from fully random processing. We further show partial evidence that piRNA biogenesis may be initiated by neighboring transposable elements.

## Introduction

A recent landmark discovery has identified a novel class of small RNAs in mammalian testes that is expressed during spermatogenesis [1–6]. PIWI-interacting RNAs (piRNAs) are typically ∼30 bases long, associate with PIWI proteins, and are organized into distinct genomic clusters (reviewed in [7–12]). The function of piRNAs is currently unknown**,** but the homology of PIWI proteins to Argonaute proteins, key components of the small interfering RNA pathway, and the similarities of piRNAs to microRNAs and short-interfering RNAs (siRNAs), known as negative regulators of gene expression, suggest a role in RNA-dependent regulatory processes during meiosis. Furthermore, piRNAs are similar to repeat-associated small interfering RNA (rasiRNA), a class of small RNAs that are responsible for transposon silencing in the *Drosophila* germline [13–20] (and recently identified in *Zebrafish* [21]), suggesting analogies between rasiRNAs and mammalian piRNAs in terms of biogenesis and function. Note that the terms rasiRNA and piRNA are often used interchangeably. Here we refer to the PIWI-interacting small RNAs from *Drosophila* and *Zebrafish* as rasiRNAs and the mammalian counterparts as piRNAs without discounting functional similarity.

**Figure 1:**
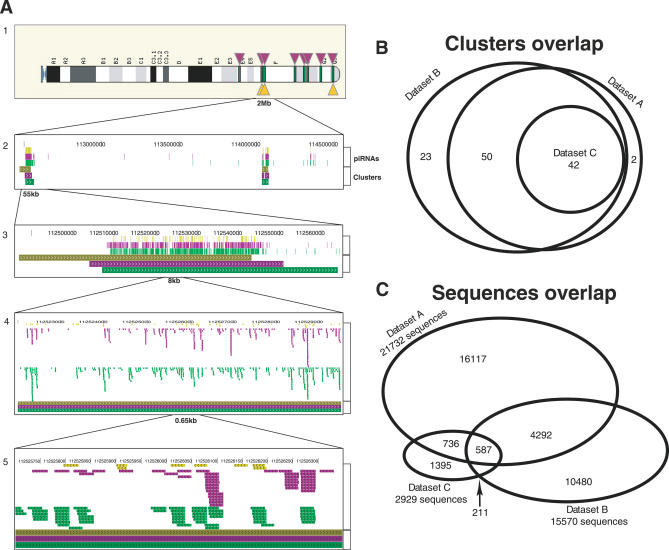
Sequence and Cluster Overlaps between Datasets A, B, and C Although the three studies identified the same piRNA clusters, they are distinct at the level of piRNA sequences. (A) View of Chromosome 5 piRNA sequences and clusters from datasets A, B, C. Top panel (1) is the karyotype view with cluster positions of the datasets: A (green lines), B (top purple triangles), and C (bottom yellow triangles). Lower panels (2–5) are magnified views of the sequences and cluster locations from the three datasets. Top three tracks in each panel are the sequence locations from datasets C (yellow), B (purple), and A (green), and lower three tracks are the cluster positions in the same color scheme. The Venn diagram of the cluster overlaps (B) shows a good agreement between the datasets while sequence overlaps, using 95% identity measure, are small (C). Note that the number of piRNAs used in this comparison is different from the number of sequences reported in the original studies (see Methods and Table S1).

To better understand the origin of piRNAs, we compared the available three largest mouse piRNA datasets (identified at the pachytene stage of spermatogenesis) in terms of sequence similarities and cluster organization. Given the comprehensive nature of these efforts and the focus on a common specific stage in mouse spermatogenesis, we expected close agreement between the datasets. Indeed, the three groups report *similar* location, size, and strand organization of the piRNA genomic clusters (Figure 1A). However, the three sets of sequences are surprisingly *dissimilar* suggesting a much larger underlying pool of potential piRNAs from which each group has been independently sampled. We estimate the size of the pool to be about ∼2 × 10^5^ potential piRNAs, based on the number of sequences in each datasets and their overlaps.

We further show that 25% of piRNA clusters are bracketed by inverted repeats of varying length, suggesting that some of the long piRNAs single-stranded precursors [1–3,6,13] can form a double-strand RNA (dsRNA) intermediate from inverted repeats that may trigger piRNA biogenesis. Taking into account positional nucleotide frequencies and copy numbers of experimentally determined piRNAs, we conclude that piRNA precursors are processed by a quasi-random mechanism that generates large numbers of distinct piRNA sequences.

### Discovery of piRNAs

Five groups reported the discovery of small RNAs expressed exclusively in mammalian testes (mouse, rat, and human) that bind MIWI (murine PIWI) or MILI proteins [1–5]. Here, we focus on the three largest datasets (A–C, listed in decreasing number of piRNA sequences identified in [1–3]) each with thousands of distinct piRNA sequences (a recent fourth comprehensive dataset of MILI-bound piRNAs identified in the pre-pachytene stage of spermatogenesis [6] is not included in this analysis). The number of unique piRNA sequences ranges from 3,482 to 40,102 (Table S1), as a result of the different methods used to identify the sequences. Overall, the length distributions of piRNAs peak at 29–31 nucleotides. However, the MILI-bound piRNAs (dataset C) [3] are generally shorter (26–28 nt) than the MIWI-bound piRNAs (29–31 nt) [1,2], possibly due to differences in binding modes of the two proteins.

The short length of piRNAs and the structural homology between PIWI and Argonaute proteins are suggestive of functional similarities between piRNAs and microRNAs. However, the combined evidence indicates that both the biogenesis and function of these two classes of RNA are distinct (Table 1). Primary differences are in genomic organization, sequence conservation, and in the number of unique sequences—among which are hundreds of microRNAs and tens of thousands of piRNAs. The majority of the identified piRNAs have a preference for a uridine base at the first position (78%–94%). Similar 5′ bias was observed in other types of small RNAs such as microRNAs and siRNAs, although to a lesser extent. The 5′ U is reminiscent of processing by RNase III enzymes [17,18] but may also reflect preferential binding to the Argonaute-like proteins. Although microRNAs and piRNAs share similar 5′ termini, other aspects of their biogenesis pathways are noticeably distinct: (i) piRNAs undergo 2′-O-methylation at their 3′ end [22–26], which animal microRNAs do not; (ii) microRNA precursors are characterized by a distinct hairpin structure whereas piRNA precursors have no apparent secondary structure; and (iii) in contrast to microRNAs, piRNA maturation is independent of Dicer enzymes [16].

**Table 1:**
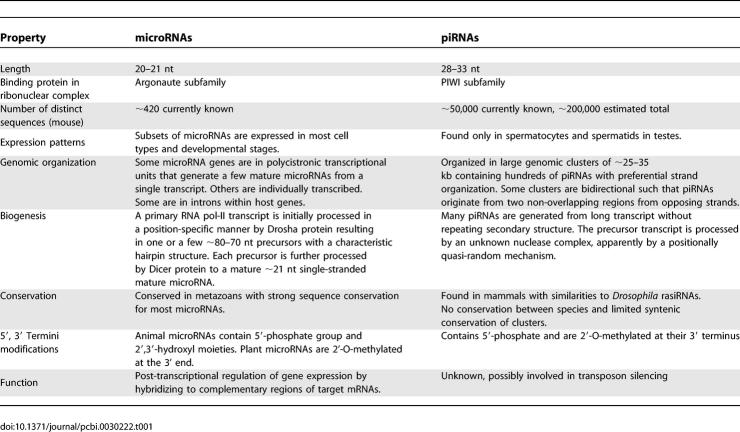
Comparison of microRNAs and piRNAs

The majority of piRNAs (81%–96%) is organized in clusters (Figure S1) with distinct strand preference that ranges from 1 to 127 kb in size and are found predominantly in autosomes. Some of the clusters are organized in a bipartite arrangement with a stretch of piRNAs on one strand adjacent to a second stretch of piRNAs on the opposing strand. This organization is consistent with bi-directional transcription—for a minority of the clusters—from a common origin that generates two RNA precursors. The organization of piRNAs into clusters is common to mouse, human, and rat with significant conservation of the cluster genomic locations (synteny) [2,3]. In contrast, there is very little conservation at the level of individual piRNA sequences (unpublished data and previously reported by [1–3,6]). Most reported piRNAs are in un-annotated intergenic regions and only a small fraction appears to be derived from mRNAs (5.7%–12%) or is coincident with other classes of RNAs such as snoRNAs, tRNAs, rRNAs, or miRNAs (0.2%–3.5%) [1–3].

piRNAs bind MILI and MIWI proteins, which are members of the PIWI protein family, a subclass of the Argonaute family. In eukaryotes, Argonaute proteins are key components of the interfering RNA pathway in which they bind mature microRNAs or siRNAs to form the RNA-induced silencing complex (RISC) [27]. All three murine PIWI members (MIWI, MILI, and MIWI2) are required for spermatogenesis as determined by knockout experiments and are predominantly expressed in testes in partially overlapping time intervals [28–31]. Recent reports link mammalian MIWI protein to chromatoid bodies (also known as nuages in *Drosophila*) [32]. These are cytoplasmic structures found in all mammalian spermatogenic cells that physically associate with the nuclear membrane during spermatogenesis and contain an RNA helicase protein (VASA). The function of chromatoid bodies is unknown but they are presumed to be the site of post-transcriptional processing and storage of mRNAs analogous to processing bodies in somatic cells (P-bodies) [33]. It is unknown if the co-localization of MIWI proteins to chromatoid bodies is linked in any way to their function with piRNAs.

### Similarities between rasiRNAs and Mammalian piRNAs

rasiRNAs are a class of interfering RNA with a size distribution of 23–28 nucleotides that were identified in a number of organisms [17]. They originate from repeat sequences related to transposable elements and heterochromatic regions [15], and evidence supports their involvement in transposon silencing [13–21]. rasiRNAs are found in both female and male germline where they bind members of the PIWI family (Piwi, Aub, and Ago3 in *Drosophila*) [13,20,34]. There are two distinct types of *Drosophila* rasiRNAs (there is evidence that similar classes exist in *Zebrafish* [21]); the first type bind Piwi or Aub proteins, are mostly antisense to transposable elements, and enriched for 5′ uridine. The second type bind Ago3 proteins, are mostly sense to the transposable elements, and enriched in adenosine at position 10. The different strand-specificity and the U and A enrichments led to the hypothesis that the biogenesis of the two types of rasiRNAs is coupled [13,20]. In this model the Piwi/Aub-associated rasiRNAs guide the 5′ cleavage of the Ago3-associated rasiRNAs by hybridization to the sense transcript. Similarly, the Ago3-bound rasiRNAs direct the 5′ cleavage of the Piwi/Aub-bound rasiRNAs by hybridization to the anti-sense transcripts. Thus, the two rasiRNA types are engaged in a mutual amplification loop that facilitates the silencing of multiple transposon copies.

The length characteristics, testis-specific expression, PIWI interaction, genomic organization, and 5′ uridine enrichment suggest that piRNAs may be the mammalian equivalent of rasiRNAs. This would support the idea that mammalian piRNAs might be involved in silencing transposable elements. However, at present, there are a number of differences that cast doubt on this functional analogy. First, genomic annotation of piRNAs indicates that only 12%–20% are repeat derived [1–3,5], which is smaller than the frequency of repeat sequences in the mouse genome (37.5%) [35], while *Drosophila* rasiRNAs originate preferentially from repeat regions. Second, mammalian piRNAs originate from one strand or the other forming clusters with continuous strand bias whereas rasiRNAs originate from both strands of the clusters with positional enrichment for “U” and “A.” We explored the analogy between rasiRNAs and piRNAs, but did not find significant 5′ partial complementarity between piRNA sequences as found in rasiRNAs [13,20]. However, at present, sequences associated with the third mouse testes–specific MIWI protein (MIWI2), also essential for spermatogenesis and linked to transposon silencing [31], have not yet been identified. Future identification of MIWI2-bound piRNAs—in analogy to Ago3-bound *Drosophila* rasiRNAs—enriched for adenosine at position 10 with partial complementary match to other piRNAs would be strongly suggestive of functional similarity between rasiRNAs and piRNAs.

### Open Questions

The discovery of large sets of piRNAs raises a number of important biological questions. In particular, what is the biochemical role and cellular function of PIWI-bound piRNAs during spermatogenesis? Are they involved in transposon silencing, chromosome rearrangements (as are 30-nt PIWI-bound RNAs in *Tetrahymena* [36,37]), or chromosome pairing? What are the evolutionary constraints on piRNA sequences? Answers to these questions will primarily emerge from further experiments. Here, we focused on the basic questions of *how many piRNA sequences* there are and *how they are produced*. We reasoned that a detailed computational comparison of the three major datasets, representing independent discoveries of piRNAs, provides insight into the organization of genomic clusters, the number and distribution of sequences within the clusters, and, by implication, their biogenesis.

## Results/Discussion

### Comparing piRNA Clusters

We first compared the cluster locations in the mouse genome from datasets A–C and found extensive agreement between the datasets. The majority of clusters overlap by more than 75% of the length of the shorter cluster. All 42 genomics clusters from dataset C, the smallest of the three, matched clusters of datasets A and B (Figure 1B). Given the different definitions of clusters in the three datasets, we conclude that the three sets of experiments have determined essentially the same clusters of piRNAs expressed in the pachytene stages of spermatogenesis (Figure S1). Other stages of development may yield additional and possibly distinct sets of piRNAs, such as the MILI-bound set of piRNAs (not analyzed here) recently identified in the pre-pachytene phase of spermatogenesis [6].

### Comparing piRNA Sequences

We compared the sets of individual sequences from the three groups (A–C). Contrary to the agreement between clusters, we found surprisingly small overlaps between the sets of unique sequences, irrespective of the criteria used for sequence comparison (100%, 95%, or 90% sequence identity, Table S1). For example, at a 95% sequence identity cutoff only 45% of the sequences from dataset C overlap with dataset A (the largest fractional overlap among all pairs of datasets), although all the piRNA clusters from the smallest dataset C are included in dataset A. Furthermore, only 587 sequences were common to all three datasets representing 20%, 3.7%, and 2.7% of the datasets C, B, and A, respectively (Figure 1C). Similarly low overlap was observed when comparing human piRNA datasets, but as the sequencing coverage is lower than in mouse, this result is not as conclusive.

### Estimating the Complete piRNA Pool

This small overlap between the piRNA datasets points to an apparent contradiction—how can different sets of piRNA sequences originate from a common set of genomic clusters? The simplest explanation is that each experiment identified only a subset of sequences from a larger pool of unique piRNA sequences. To quantify this effect, we first asked whether the observed overlaps are within the expected range assuming that the complete piRNA pool is simply the union of the three datasets. To facilitate the comparison we restricted this analysis to the intersection of clusters from the three datasets, termed “intersection clusters” (Table S2). By numerical simulation and direct calculation we find that the observed sequence overlaps between all datasets is significantly lower than expected (unpublished data), indicating that the total pool of piRNA sequences is indeed larger than the simple union of current datasets. Using straightforward statistical calculation, we then estimated the total number of piRNAs from the observed overlaps in the intersection clusters by considering the three studies as independent sampling experiments from a common pool of all piRNAs (Figure S2).

**Figure 2:**
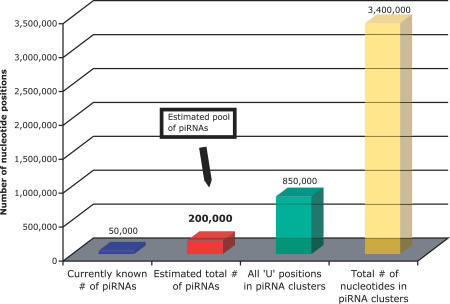
Currently Known and Estimated Total Number of piRNAs We estimate that the total number of piRNAs in mouse testes is ∼2 × 10^5^ (red), roughly four times the number of currently known piRNAs (blue). The estimated number of piRNAs corresponds to ∼23% of all “U” positions (green) or 5%–6% of all nucleotides (yellow) in piRNA clusters.

From this estimate we conclude that the current datasets analyzed here have so far identified only 25%–30% of all potential piRNA sequences from the pachytene stage of mouse spermatogenesis. This implies that in the complete set ∼20%–25% of all “U” positions in the clusters are potential start sites for piRNA sequences when taking into account the pronounced preference for 5′ uridine. Extrapolating to saturation in all clusters reported by any of the three groups, we arrive at the overall conservative estimate of *N_total_* ≈ 2 × 10^5^ potential piRNA sequences in mouse testes (Figure 2). This does not imply that all sequences are necessarily present in any given cell.

### Quasi-Random Processing

The details of piRNA biogenesis are not yet known. In particular, what is the precursor form of piRNAs? Is it single-strand or double-strand? What are the components of the nuclease-processing complex? By which mechanism, in which order, and under which regulatory control do thousands of different ∼30 nt transcripts originate from a limited number of genomic regions? The large differences in piRNA datasets and the relatively weak evolutionary conservation of piRNA sequences suggest that the processing of piRNAs from a primary precursor is not a precise step, in contrast to microRNA maturation. Instead, it appears, to a first approximation, that piRNAs are generated by a random mechanism in which any U position is a potential 5′ piRNA start. This notion is supported by the fact that sequence overlap between the datasets remains low even when we compare only the more abundant sequences (Figure S3), and that there is no evidence for repetitive spacing between consecutive sequences (unpublished data). However, there appears to be some non-randomness in that some positions are preferentially processed into piRNAs (see patterns in Figure 1A, panels 4 and 5). In particular, a sizable fraction (∼20%) of all piRNA sequences were cloned three or more times, and we find that many piRNA sequences from the same strand are partially overlapping (Figure S4). This suggests some, albeit weak, sequence effects within a genomic cluster, either at the level of nuclease processing or at the level of loading into a PIWI complex. We use the term “quasi-random” to reflect this weak departure from random processing.

### Weak Discriminating Sequence Motif

**Figure 3:**
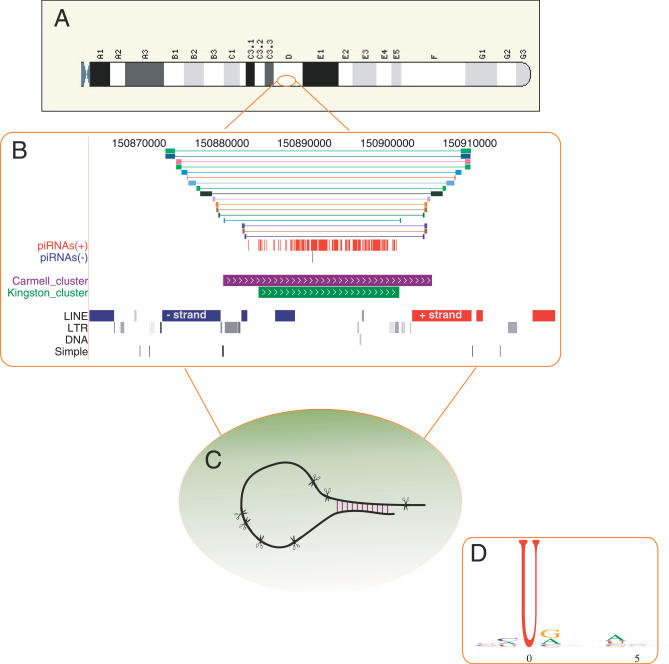
A Model of piRNA Biogenesis piRNAs originate from long RNA precursors transcribed from a small number of genomic regions (A). Some clusters contain inverted repeats that can potentially form dsRNA fold-back structures. In this genomic view of a cluster chr2: 150870000–150910000 (B), the inverted repeats are represented as linked colored bars. These inverted repeats originate from inverted LINE transposable elements that flank the piRNA cluster (red and blue bars in the LINE track). A long transcript containing the pair of inverted LINE elements can potentially form a precursor with a dsRNA segment (C). piRNAs are processed by a quasi-random mechanism with a weak sequence preference near the 5′ U that is most pronounced in frequent clones (D).

We therefore attempted to identify a distinguishing sequence signal that predicts which U bases are 5′ piRNA cleavage sites. Using a sequence classification algorithm, we identified, with 61% accuracy, the correct 5′ U piRNA sites from all other U positions using both 10-fold cross-validation on the training set and by testing on randomly withheld test set excluded from training (see Methods). Although the classification accuracy is low, it is significantly better than random prediction (classification on randomized data did not exceed 50%). Furthermore, the classification accuracy improved to 72% when the algorithm was trained and tested on the abundant piRNA sequences (clone counts >2). The differentiating signal is a weak preference for a G or A in the +1 position (relative to the 5′ U), an A in the +4 position, and a slight under-representation of G at the −1 position (Figure 3). These results suggest that the processing of the precursor is quasi-random in that there is a weak yet significant non-random sequence preference at the 5′ cleavage site.

### Model of piRNA Biogenesis

The precursor form of piRNA primary transcript— single- or double-stranded—is currently unknown. However, the strong 5′ uridine bias and the presence of the 5′ phosphate group [4] in mature piRNAs is indicative of a dsRNA precursor that is processed by an RNase III type enzyme [3], although no such nuclease has so far been implicated in piRNA processing, and piRNA processing is independent of Dicer [9].

In Caenorhabditis elegans, germline silencing of transposable elements by the RNAi pathway is initiated by a dsRNA structure formed by base pairing of the terminal inverted repeats of the transposon in a fold-back structure [38]. To investigate whether a similar mechanism may be involved in piRNA biogenesis, we searched for inverted repeats in or near the vicinity of piRNA clusters. Such inverted repeats may form precursors containing dsRNA that initiate enzymatic processing. Overall, we found that 63% of all clusters have inverted repeats of length 100 bases or longer (see Methods) and that 25% of all clusters are bracketed by inverted repeats, i.e., the complementary segments are at the ends of the clusters (Figure 3B). Surprisingly, some of the flanking inverted repeats coincide with inverted transposable elements such as SINEs, LINEs, and LTRs that are on opposite strands, one on each side of the cluster (Figures 3B and S5), suggesting a link between transposable elements and piRNA biogenesis.

Recent studies propose that mammalian piRNAs may be involved in transposon silencing analogous to *Drosophila* rasiRNAs, although the mechanistic details remain to be determined [6,13]. The model of transposon silencing by rasiRNAs put forward by [13,20] explains the feed-forward amplification of the silencing process but not its initiation. They propose that the induction requires a pool of initiating rasiRNAs that triggers a mutual amplification loop between the Ago3-bound and the Piwi/Aub-bound rasiRNAs. The source of the initiating rasiRNAs is unknown, and they may be maternally inherited by the developing oocytes.

We hypothesize that one plausible model of piRNA biogenesis involves long transcripts that contain flanking inverted transposable elements, one at each end of the cluster (Figure 3B). Such precursors can arise, for example, by continuous transcription of one of the repeats past its termination site. If the transcript reaches the other end of the cluster and includes the sequence complementary to the repeat element on the opposing strand, the transcript can potentially form a dsRNA segment. piRNA biogenesis is then triggered by processing of the dsRNA segments which generate the initiating pool of piRNAs. Similar to the *Drosophila* model of rasiRNA generation [13,20], these initial sequences may act on transcripts derived from other locations (in trans) containing at least one copy of the initiating repeat element and resulting in the production of a much larger pool of piRNAs.

We cannot exclude the possibility that the bracketing inverted transposable elements are not part of the primary transcript but simply the result of statistical coincidence. In fact, similar numbers of such repeats are found in randomly chosen genomic regions (unpublished data), as remnants of transposable elements account for over a third of the mouse genome [35], but most of these may not be expressed. In contrast, the bracketing inverted repeat structures must be transcriptionally active, and we do find that a number of the transposable elements near piRNA clusters are indeed expressed in testes (as indicated by ESTs recorded in genome databases). Alternatively to the initiating dsRNA structure, a single-strand RNA precursor may be a direct substrate of a nuclease, yet to be discovered, that generates approximately 30-residue long 5′ P products.

### Conclusions

The novel discovery of piRNAs has extended the multifaceted family of small interfering RNAs that includes microRNAs, siRNAs, and rasiRNAs. The tens of thousands of distinct mouse piRNAs observed so far map to ∼117 distinct genomics locations in the genome. The details of piRNA transcriptional control, such as promoter sites and transcription factors, remain to be determined. Our analysis has revealed low sequence overlap between the currently known pachytene-stage mouse piRNA datasets, although the sequences originate from a common set of genomic clusters. This apparent contradiction is resolved by noting lack of saturation in each individual experiment. We interpret the low sequence overlap as suggestive of quasi-random sub-saturation processing from common precursors, such that different experiments yield different and only partially overlapping sets of piRNAs. In addition, based on the observation of repeat structures bracketing some of the clusters, we propose that one plausible mechanism for initiation of piRNA biogenesis involves long transcripts with terminal inverted repeats, possibly derived from (remnants of) transposable elements. Such transcripts may form partial dsRNA intermediates initiating enzymatic degradation. Subsequent stages of piRNA biogenesis may then follow the ping-pong model proposed by [13,20].

The notion that piRNAs both direct the degradation and are the degradation products of their own precursors suggests that piRNA transcripts are under strict regulation at a crucial stage of meiosis. What is their function? The PIWI proteins are highly expressed in the pre-pachytene and pachytene stages of meiosis when chromosome pairing is completed (zygotene) and synapsis is peaked. This raises the intriguing possibility that the transcripts from which the piRNAs derive, and/or the piRNAs themselves, are involved in one of the crucial processes of meiosis, correct chromosome pairing, for which the molecular mechanism remains a mystery. The connection between this and the proposed piRNA function of transposon silencing remains to be elucidated. We look forward to directed biochemical and genomic experiments that will invalidate or confirm the models proposed here and explain the function of piRNAs.

## Methods

### Datasets.

Mouse piRNA sequences were collected from the following sources: Dataset A from Lau et al. [1]; Table S4 contains 65,535 unique small RNA sequences. After removing known small RNA sequences, the remaining 40,102 were considered as piRNA sequences for this dataset. Dataset B from Girard et al.[2] (personal communication) includes 51,331 reads representing 28,956 unique sequences. Dataset C from Aravin et al. [3] (Table 4 therein) contains 5,444 small RNA sequences of which 3,482 are unique sequences annotated as piRNAs. Dataset D from Watanabe et al. [4] (Table S7 therein) contains 357 unique small RNA sequences. Dataset E from Grivna et al. [5] (Table S1 therein) contains 40 unique sequences.

Duplicate and subsequences were removed from each dataset at 100% nucleotide identity (Table 1). In cases where genomic annotation was provided we removed known small RNAs (miRNAs, tRNAs, and snoRNAs) as well as apparent rRNA and mRNA fragments from the dataset. All sequences and clusters were mapped to mouse genome build mm7 (August 2005) taking the best genomic match up to a maximum of two mismatches or gaps. Sequence matching to the genome was performed using a combination of WU-BLAST (http://blast.wustl.edu/) and our in-house alignment software developed jointly with M. Zavolan. The following BLAST arguments were used for short sequence alignments:

−W = 6 − X = 50 − gapX = 50 − S2 = 50 − gapS2 = 50 − hspmax = 1,000 − gspmax = 1,000 − E = 1,000 − filter = none.

Over 90% of the sequences mapped to unique genomic locations. In the remaining cases where there was more than one match to the genome, all positions were considered as a possible origin of the piRNA.

Coordinates of piRNA clusters from dataset C were translated from mm6 to mm7, in some cases resulting in a change in cluster length due to partial mapping:

cluster3 : mm6|chr9|+|67822641|67883254; mm7|chr9|+|67751406|67785923
cluster8 : mm6|chr14|+|22446408|22484616; mm7|chr14|+|21745838|21783387
cluster15 : mm6|chr9|+|54305216|54360650; mm7|chr9|+|54231430|54253257
cluster19 : mm6|chr17|+|63838569|63952874; mm7|chr17|+|64406371|64449447

The datasets were not significantly biased to specific sequences or nucleotide composition by experimental protocol. The two larger datasets (A and B) were produced using similar ligation adaptors and sequencing methods excluding the possibility of sequence bias due to different methodologies. Indeed, we found no differences in mononucleotide or dinucleotide frequencies between the datasets.

### Comparison of genomic clusters and definition of intersection clusters.

Overlaps between genomic clusters from different datasets were determined by intersection of their genomic locations. The length of the overlaps ranged from 19% to 100% of the shorter cluster. In the majority (70%) of the overlapping clusters, the extent of the overlap covered >75% of the length of the shorter cluster. Instances where two clusters from one dataset overlapped a single cluster from another dataset were counted as one overlap. Intersection clusters were defined as the genomic regions where clusters from all three datasets overlapped (See Table S2).

### Sequence comparison.

Sequence comparison was performed as follows: All sequences (after initial processing) from all datasets were combined and compared all-against-all using WU-BLAST and in-house software. Sequences were grouped into similarity sets by hierarchical clustering and a defined identity measure. To explore sensitivity of the analysis to variation in parameters, we performed three clustering procedures using these identity measures: (i)100% sequence identity over the entire length of the shortest sequence; (ii) 95% sequence identity over 95% length of the shortest sequence; and (iii) 90% sequence identity over 90% length of the shortest sequence. Considering all sequences in a similarity cluster to be essentially identical, the degree of overlap between two datasets is determined by counting the number of similarity clusters that contain sequences from both datasets (Table S1). Similarly, the three-way overlap between datasets A, B, and C was determined by counting the number of similarity clusters that contained sequences from all three groups (Figure 1C). The comparison of the abundant piRNA sequences (higher clone counts) was performed in the same way using only sequences that were cloned >2 times (Figure S3).

Human piRNA sequences were retrieved from Girard et al. (dataset B) and Aravin et al. (dataset C) studies. Similarly to mouse piRNAs, sequences that matched known small RNAs and mRNAs were removed resulting in 9,600 unique piRNA sequences from dataset B and 120 sequences from dataset C. Sequences comparison was performed as outlined above. Under 95% sequence identity measure, 29 sequences were shared between the two datasets corresponding to ∼24% of dataset C sequences.

### Estimate of the total number of piRNAs.

The degree of overlap between two independent datasets, say *X* and *Y*, in a genomic intersection cluster is modeled by a hypergeometric distribution with a mean 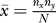 where *n_x_* and *n_y_* are the number of piRNA sequences in the cluster in datasets X and Y, respectively, and *N* is the total number of piRNAs in the cluster, which is unknown. This corresponds to random selection of *n_x_* and *n_y_* piRNA sequences from a total pool of *N* unique sequences, i.e., ignoring varying clone counts. Under the maximum likelihood assumption, the observed overlap between the two datasets is the most likely value. That is, 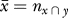 where *n_x_*_ ∩ *y*_ is the size of the observed overlap between datasets *X* and *Y*. For the purpose of this approximation, the size of the overlap *n_x_*_ ∩ *y*_ was determined by a 95% sequence identity criterion (see above).

The value of total number of piRNAs *N* can then be computed directly as:

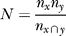



For each intersection cluster we computed three estimates of *N*: *N_AB_*,*N_AC_*,*N_BC_* based on the observed overlaps between datasets AB, AC, and BC (Figure S2).

The total number of piRNAs was computed as the average of the three approximations summed over all clusters:

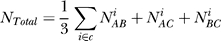
where *i* is an intersection cluster, *c* is the set of all intersection clusters, 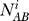 is the computed total number of piRNAs in cluster *i* based on the overlap between datasets A and B, and similarly for 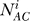 and 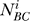 .

To approximate the total number of piRNAs in the mouse genome we extrapolated the total number in all intersection clusters, to the union of all clusters from datasets A, B, and C (Table S3), by multiplying *N_Total_* by the ratio of the combined length of the union of all clusters to the combined length of all intersection clusters (Figure S2).

### piRNA distance distribution.

Sequences assigned to genomic positions were sorted by chromosomal position. The distance between two adjacent sequences *i,j* mapped to the *same* strand is determined by:

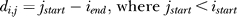
When i and j are overlapping *d_i_*_,*j*_ ≤ 0.

### Support vector machine classification of 5′ U piRNA sites.

To identify a distinguishing signal for 5′ piRNA processing in cluster regions, we trained a support vector machine classifier to discriminate between 5′ piRNA and all other uridine positions.

Positive set included all of the piRNAs 5′ uridine positions extended ten bases upstream and downstream; a total of 24,604 sequences. Similarly, the negative set was constructed by selecting random non-piRNA uridine positions in the intersection clusters and ten nucleotides upstream and downstream. Both sets were split into two, one part used for training and the other for testing. Feature vectors were constructed by converting the 21-base sequences into 84-bit vectors (21 nt × 4 bases), i.e., each nucleotide position is converted to a 4-bit vector representing the RNA base.

Support vector machine training and classification was performed using an R interface of “libsvm” (http://cran.r-project.org/src/contrib/Descriptions/e1071.html) using a polynomial kernel of degree 3. Classification accuracy in a 10-fold cross-validation on the training set and testing procedure on an independent test set was ∼61%, whereas classification using a randomized training set did not exceed 50% accuracy. Using the high frequency piRNAs (cloned >2 times) as the positive training set, the prediction accuracy in 10-fold cross-validation and with the test set improves to 72%. In a feature selection process we found that positions −1, +1, and +4 (relative to the starting uridine position 0) were the largest contributors to the classification (Figure 3D). Information content analysis revealed a preference for G or A in positions +1, for an A in positions +4, and under-representation of G at position −1.

### Inverted repeats analysis.

For detection of inverted repeats in the vicinity of cluster, sequences were collected from the union clusters (Table S3) and extended by 10 kb in both 5′ and 3′ directions. The sequences were aligned to their complements by “bl2seq” (a BLAST implementation for aligning two sequences) in gapless mode (using –g F flag). Alignments longer than 100 bases with >90% identity were mapped to the mouse genome and used in subsequent analysis.

## Supporting Information

Figure S1 piRNA Clusters in the Mouse Genome (100 KB PDF)

Figure S2 Estimation of the Number of piRNAs in Intersection Clusters (1.2 MB PDF)

Figure S3 Sequence Overlap of Abundant piRNAs (18 MB PDF)

Figure S4 Distribution of Spacing between Consecutive piRNA Sequences (66 KB PDF)

Figure S5 Inverted Repeats Derived from Transposable Bracketing piRNA Clusters (60 KB PDF)

Table S1 Sequence Overlap between piRNA Datasets (19 KB XLS)

Table S2 Genomic Positions of Intersection Clusters (22 KB XLS)

Table S3 Genomic Positions of Union Clusters (31 KB XLS)

## Abbreviations

dsRNA: double-strand RNA
MIWI: murine PIWI
piRNA: PIWI-interacting RNA
rasiRNA: repeat-associated small interfering RNA
siRNA: small interfering RNA

## References

[pcbi-0030222-b001] Lau NC, Seto AG, Kim J, Kuramochi-Miyagawa S, Nakano T (2006). Characterization of the piRNA complex from rat testes. Science.

[pcbi-0030222-b002] Girard A, Sachidanandam R, Hannon GJ, Carmell MA (2006). A germline-specific class of small RNAs binds mammalian Piwi proteins. Nature.

[pcbi-0030222-b003] Aravin A, Gaidatzis D, Pfeffer S, Lagos-Quintana M, Landgraf P (2006). A novel class of small RNAs bind to MILI protein in mouse testes. Nature.

[pcbi-0030222-b004] Watanabe T, Takeda A, Tsukiyama T, Mise K, Okuno T (2006). Identification and characterization of two novel classes of small RNAs in the mouse germline: retrotransposon-derived siRNAs in oocytes and germline small RNAs in testes. Genes Dev.

[pcbi-0030222-b005] Grivna ST, Beyret E, Wang Z, Lin H (2006). A novel class of small RNAs in mouse spermatogenic cells. Genes Dev.

[pcbi-0030222-b006] Aravin AA, Sachidanandam R, Girard A, Fejes-Toth K, Hannon GJ (2007). Developmentally regulated piRNA clusters implicate MILI in transposon control. Science.

[pcbi-0030222-b007] Kim VN (2006). Small RNAs just got bigger: Piwi-interacting RNAs (piRNAs) in mammalian testes. Genes Dev.

[pcbi-0030222-b008] O'Donnell KA, Boeke JD (2007). Mighty Piwis defend the germline against genome intruders. Cell.

[pcbi-0030222-b009] Zamore PD (2007). RNA silencing: genomic defense with a slice of pi. Nature.

[pcbi-0030222-b010] Lin H (2007). piRNAs in the germline. Science.

[pcbi-0030222-b011] Seto AG, Kingston RE, Lau NC (2007). The coming of age for Piwi proteins. Mol Cell.

[pcbi-0030222-b012] Hartig JV, Tomari Y, Forstemann K (2007). piRNAs: The ancient hunters of genome invaders. Genes Dev.

[pcbi-0030222-b013] Brennecke J, Aravin AA, Stark A, Dus M, Kellis M (2007). Discrete small RNA-generating loci as master regulators of transposon activity in Drosophila. Cell.

[pcbi-0030222-b014] Pelisson A, Sarot E, Payen-Groschene G, Bucheton A (2007). A novel repeat-associated small interfering RNA-mediated silencing pathway downregulates complementary sense gypsy transcripts in somatic cells of the Drosophila ovary. J Virol.

[pcbi-0030222-b015] Saito K, Nishida KM, Mori T, Kawamura Y, Miyoshi K (2006). Specific association of Piwi with rasiRNAs derived from retrotransposon and heterochromatic regions in the Drosophila genome. Genes Dev.

[pcbi-0030222-b016] Vagin VV, Sigova A, Li C, Seitz H, Gvozdev V (2006). A distinct small RNA pathway silences selfish genetic elements in the germline. Science.

[pcbi-0030222-b017] Aravin AA, Lagos-Quintana M, Yalcin A, Zavolan M, Marks D (2003). The small RNA profile during Drosophila melanogaster development. Dev Cell.

[pcbi-0030222-b018] Chen PY, Manninga H, Slanchev K, Chien M, Russo JJ (2005). The developmental miRNA profiles of zebrafish as determined by small RNA cloning. Genes Dev.

[pcbi-0030222-b019] Megosh HB, Cox DN, Campbell C, Lin H (2006). The role of PIWI and the miRNA machinery in Drosophila germline determination. Curr Biol.

[pcbi-0030222-b020] Gunawardane LS, Saito K, Nishida KM, Miyoshi K, Kawamura Y (2007). A slicer-mediated mechanism for repeat-associated siRNA 5′ end formation in Drosophila. Science.

[pcbi-0030222-b021] Houwing S, Kamminga LM, Berezikov E, Cronembold D, Girard A (2007). A role for Piwi and piRNAs in germ cell maintenance and transposon silencing in Zebrafish. Cell.

[pcbi-0030222-b022] Saito K, Sakaguchi Y, Suzuki T, Suzuki T, Siomi H (2007). Pimet, the Drosophila homolog of HEN1, mediates 2′-O-methylation of Piwi- interacting RNAs at their 3′ ends. Genes Dev.

[pcbi-0030222-b023] Horwich MD, Li C, Matranga C, Vagin V, Farley G (2007). The Drosophila RNA methyltransferase, DmHen1, modifies germline piRNAs and single-stranded siRNAs in RISC. Curr Biol.

[pcbi-0030222-b024] Kirino Y, Mourelatos Z (2007). The mouse homolog of HEN1 is a potential methylase for Piwi-interacting RNAs. RNA.

[pcbi-0030222-b025] O'Hara T, Sakaguchi Y, Suzuki T, Ueda H, Miyauchi K (2007). The 3′ termini of mouse Piwi-interacting RNAs are 2′-O-methylated. Nat Struct Mol Biol.

[pcbi-0030222-b026] Kirino Y, Mourelatos Z (2007). Mouse Piwi-interacting RNAs are 2′-O-methylated at their 3′ termini. Nat Struct Mol Biol.

[pcbi-0030222-b027] Carmell MA, Xuan Z, Zhang MQ, Hannon GJ (2002). The Argonaute family: Tentacles that reach into RNAi, developmental control, stem cell maintenance, and tumorigenesis. Genes Dev.

[pcbi-0030222-b028] Deng W, Lin H (2002). Miwi, a murine homolog of piwi, encodes a cytoplasmic protein essential for spermatogenesis. Dev Cell.

[pcbi-0030222-b029] Kuramochi-Miyagawa S, Kimura T, Ijiri TW, Isobe T, Asada N (2004). Mili, a mammalian member of piwi family gene, is essential for spermatogenesis. Development.

[pcbi-0030222-b030] Sasaki T, Shiohama A, Minoshima S, Shimizu N (2003). Identification of eight members of the Argonaute family in the human genome. Genomics.

[pcbi-0030222-b031] Carmell MA, Girard A, van de Kant HJ, Bourc'his D, Bestor TH (2007). MIWI2 is essential for spermatogenesis and repression of transposons in the mouse male germ line. Dev Cell.

[pcbi-0030222-b032] Kotaja N, Bhattacharyya SN, Jaskiewicz L, Kimmins S, Parvinen M (2006). The chromatoid body of male germ cells: Similarity with processing bodies and presence of Dicer and microRNA pathway components. Proc Natl Acad Sci U S A.

[pcbi-0030222-b033] Parvinen M (2005). The chromatoid body in spermatogenesis. Int J Androl.

[pcbi-0030222-b034] Nishida KM, Saito K, Mori T, Kawamura Y, Nagami-Okada T (2007). Gene silencing mechanisms mediated by Aubergine piRNA complexes in Drosophila male gonad. RNA.

[pcbi-0030222-b035] Waterston RH, Lindblad-Toh K, Birney E, Rogers J, Abril JF (2002). Initial sequencing and comparative analysis of the mouse genome. Nature.

[pcbi-0030222-b036] Mochizuki K, Fine NA, Fujisawa T, Gorovsky MA (2002). Analysis of a piwi-related gene implicates small RNAs in genome rearrangement in tetrahymena. Cell.

[pcbi-0030222-b037] Lee SR, Collins K (2006). Two classes of endogenous small RNAs in Tetrahymena thermophila. Genes Dev.

[pcbi-0030222-b038] Sijen T, Plasterk RH (2003). Transposon silencing in the Caenorhabditis elegans germ line by natural RNAi. Nature.

