# Supplementary material for: Computational Analysis of Mouse piRNA Sequence and Biogenesis
Source: PLoS Comput Biol. 2007 Nov 9;3(11):e222. doi: 10.1371/journal.pcbi.0030222 (PMC2065894; doi:10.1371/journal.pcbi.0030222)

Supplementary figure 1

Supplementary figure 1. piRNA clusters in the mouse genome. The location of piRNA clusters in the mouse genome are indicated by data source: orange lines for dataset C, magenta triangles on the left for dataset B and green triangles on the right for dataset A. There are roughly 115 clusters ranging from 1-127 kb restricted primarily to autosomes. There is good agreement of cluster positions between the datasets and the majority of the clusters overlap in >75% of their length suggesting that most of the piRNA clusters in the mouse genome are identified.

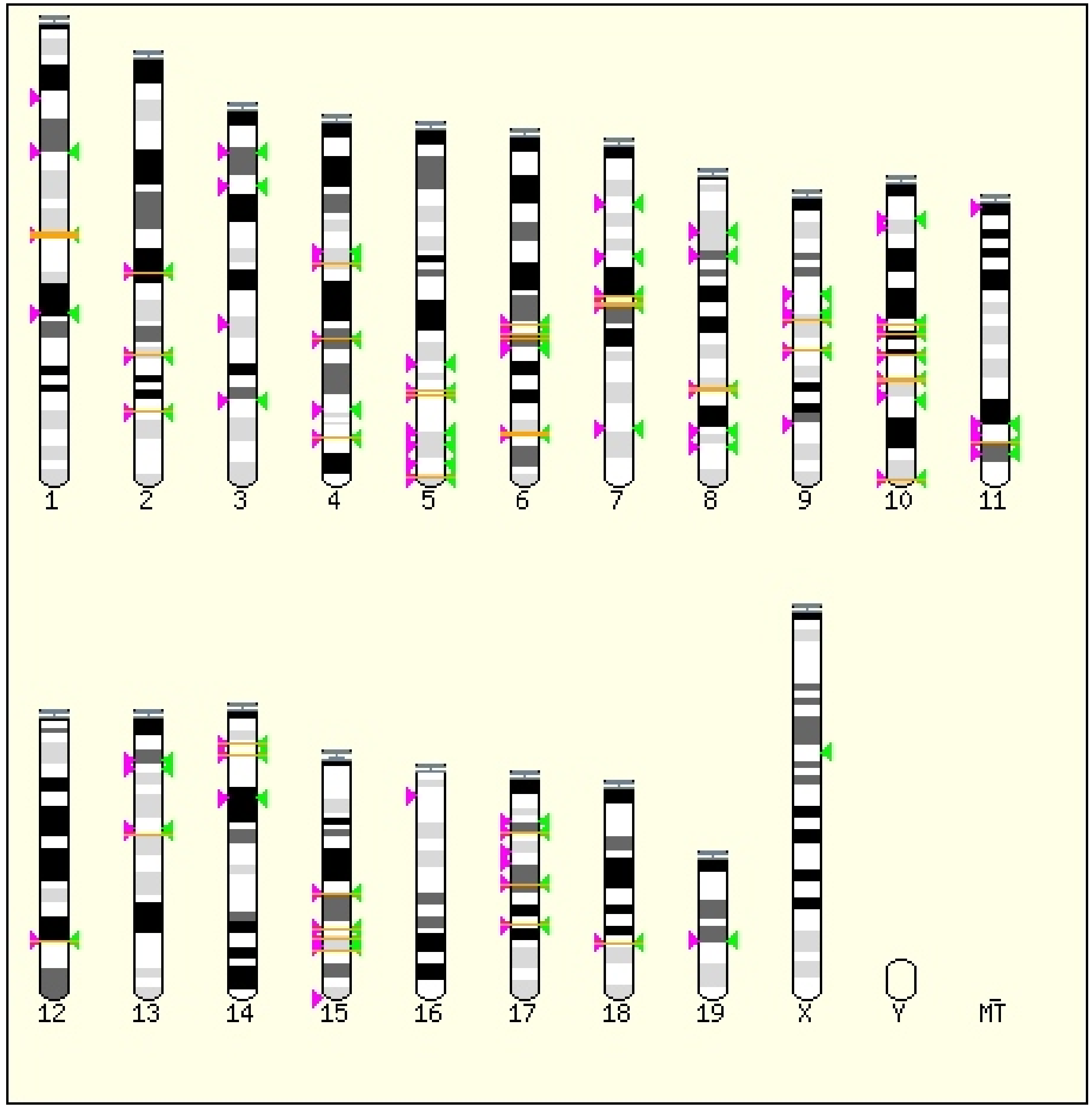

Supplement: Supplementary file 1 [file pcbi.0030222.sg001.pdf]
