# Supplementary material for: Computational Analysis of Mouse piRNA Sequence and Biogenesis
Source: PLoS Comput Biol. 2007 Nov 9;3(11):e222. doi: 10.1371/journal.pcbi.0030222 (PMC2065894; doi:10.1371/journal.pcbi.0030222)
Supplement: Supplementary file 2 [file pcbi.0030222.sg002.pdf]

Supplementary figure 2

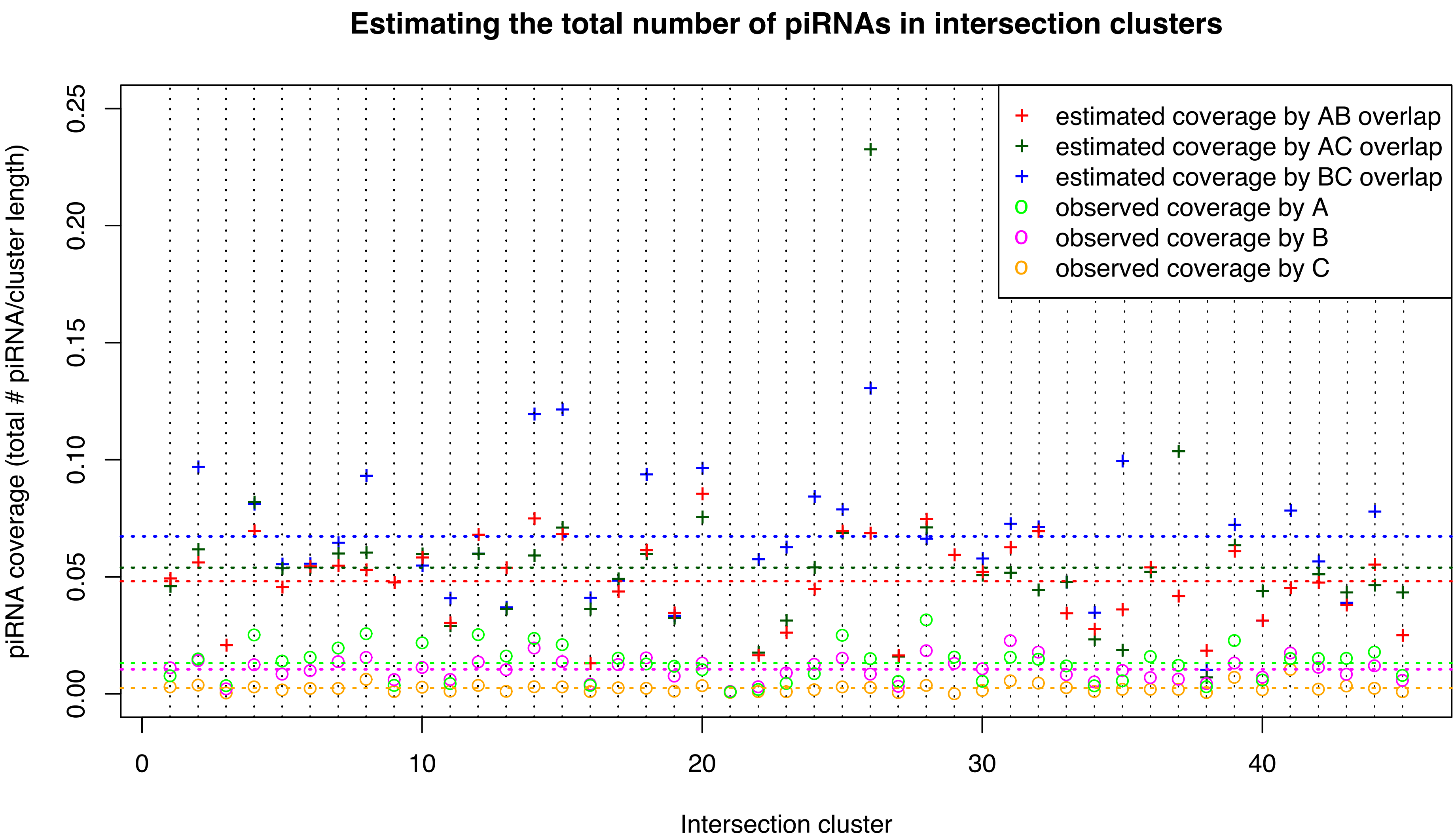

Supplementary figure 2. Estimation of the number of piRNAs in intersection clusters. A graph of the predicted and current number of piRNAs in the 45 intersection clusters (cluster intersection of datasets A, B, C). For graphing purposes the number of piRNAs in each cluster is divided by the length of the cluster representing piRNA coverage. Circles represent the current piRNA coverage by each of the datasets (A, green, B, magenta, C, orange). The cross markers represent the predicted piRNA coverage as estimated from the three intersections of the three datasets : A-B. A-C, B-C. Horizontal lines are the mean of each dataset or approximation. The estimate suggests that 5%-6% of the cluster positions are potential sites of piRNAs corresponding to ~190,000 (+/- 30,000) piRNAs in the mouse genome.
