# Supplementary material for: Computational Analysis of Mouse piRNA Sequence and Biogenesis
Source: PLoS Comput Biol. 2007 Nov 9;3(11):e222. doi: 10.1371/journal.pcbi.0030222 (PMC2065894; doi:10.1371/journal.pcbi.0030222)
Supplement: Supplementary file 3 [file pcbi.0030222.sg003.pdf]

Supplementary figure 3

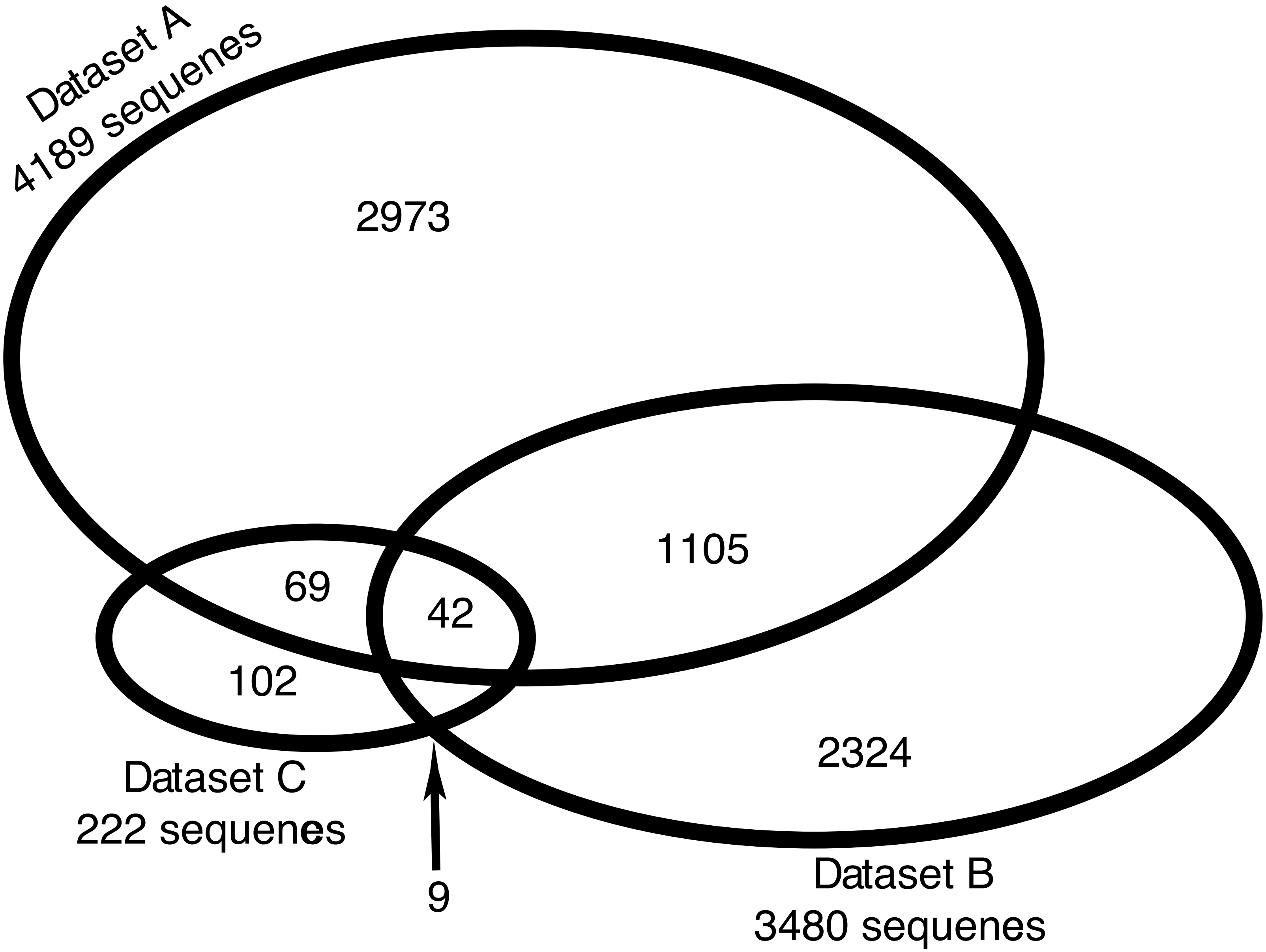

Supplementary figure 3. **Sequence overlap of abundant piRNAs**. Venn diagram of overlap between the datasets using piRNA sequences that were cloned three or more times. Sequences similarity was determined by a 95% identity measure. The degree of overlap is similar to the general piRNA population (Fig. 1) suggesting that the abundant piRNAs from different datasets do not originate from the same genomic locations.
