# Supplementary material for: Computational Analysis of Mouse piRNA Sequence and Biogenesis
Source: PLoS Comput Biol. 2007 Nov 9;3(11):e222. doi: 10.1371/journal.pcbi.0030222 (PMC2065894; doi:10.1371/journal.pcbi.0030222)
Supplement: Supplementary file 4 [file pcbi.0030222.sg004.pdf]

Supplementary figure 4

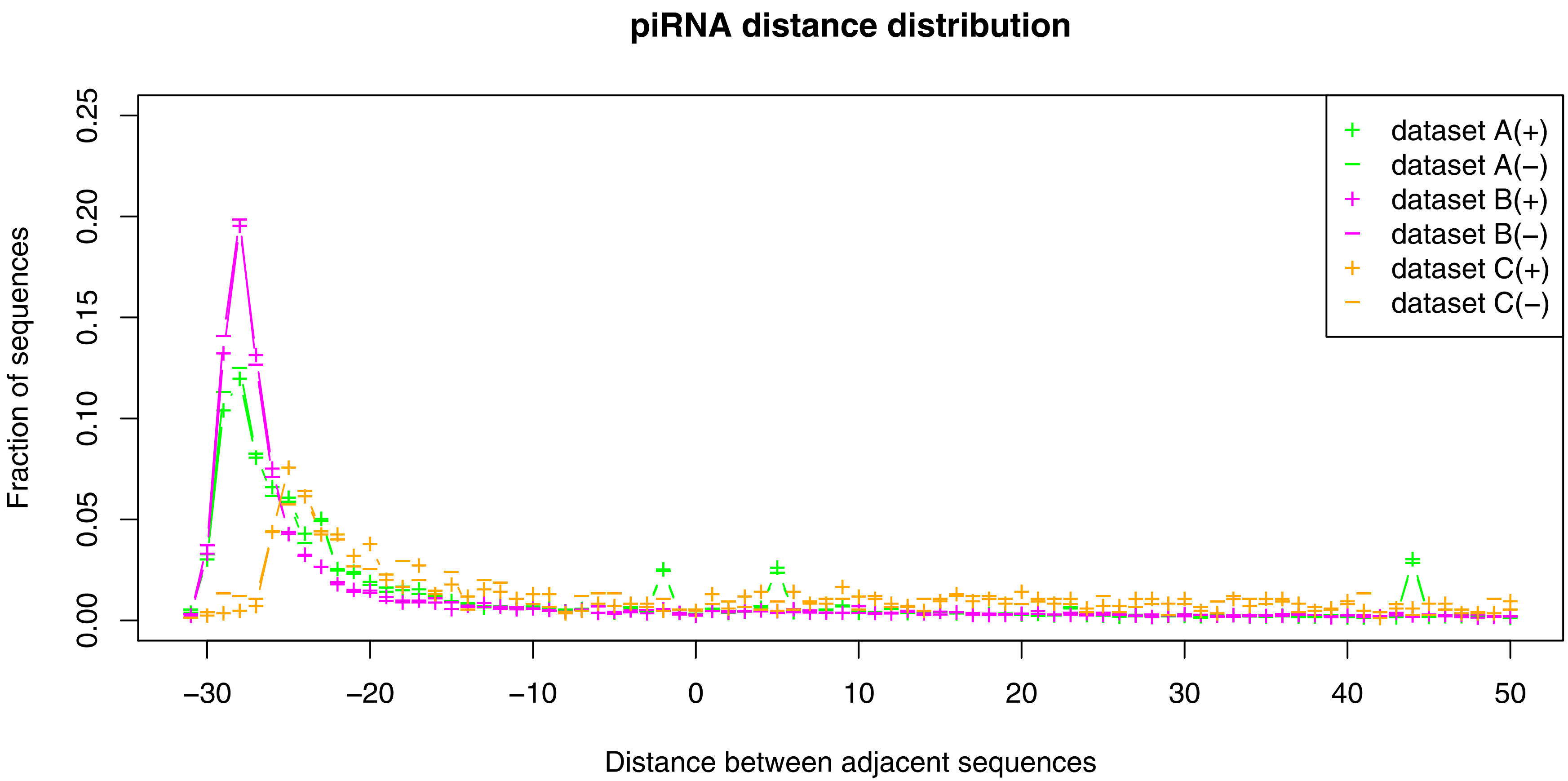

Supplementary figure 4: Distribution of spacing between consecutive piRNA sequences. A graph of the distribution of spacing between adjacent sequences measured as the distance from the end of piRNA to the start of the next sequence in the same cluster. Note that the plot is truncated at 50 nt although a small fraction of sequences are at distance >50. The spacing distribution is plotted separately for the positive (+) and negative (-) strands for each dataset. The distribution for dataset C is shifted slightly towards the larger distances reflecting the lower number of piRNA sequences that were identified relative to larger datasets A and B. Negative distances represent overlapping sequences. The large number of overlapping sequences suggests that piRNAs are not uniformly distributed in the clusters, possibly indicating a substructure organization within the genomic clusters.
