# Supplementary material for: Computational Analysis of Mouse piRNA Sequence and Biogenesis
Source: PLoS Comput Biol. 2007 Nov 9;3(11):e222. doi: 10.1371/journal.pcbi.0030222 (PMC2065894; doi:10.1371/journal.pcbi.0030222)
Supplement: Supplementary file 5 [file pcbi.0030222.sg005.pdf]

## Supplementary figure 5

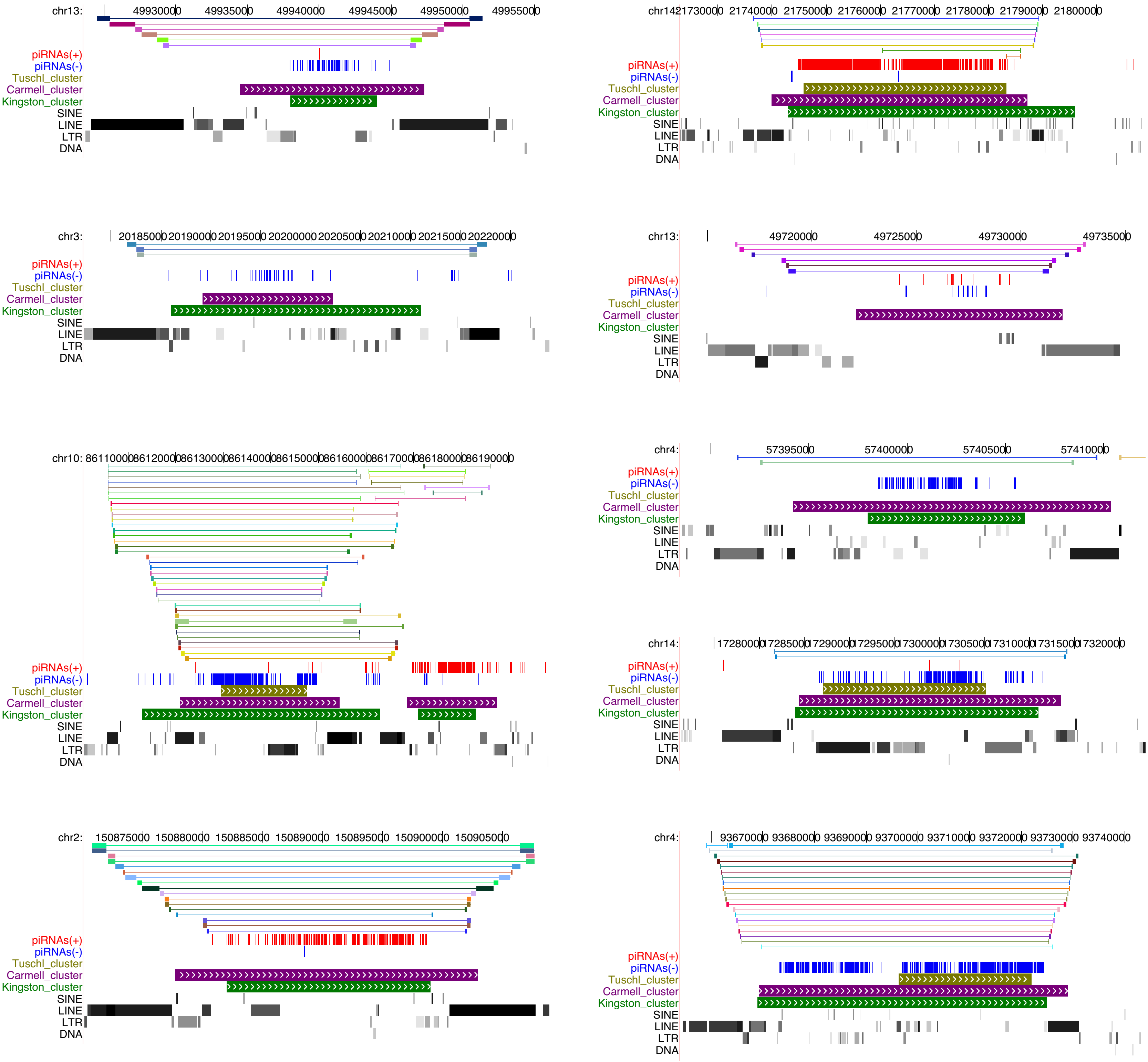


**Supplementary figure 5**: **Inverted repeats derived from transposable elements bracketing piRNA clusters.** A subset of the piRNA clusters have inverted repeats that are similar in sequence to transposable elements. In each panel view (from top to bottom): genomic coordinates; pairs of inverted sequences (block segments connected by colored lines); piRNA sequences (red and blue tracks for the + and - strands, respectively); piRNA clusters from each of the three datasets (long colored bars); SINE, LINE, LTR and DNA repeat elements (black and grey tracks). The overlap between the transposable elements and the inverted repeats indicates that the flanking transposable elements are in opposite orientation. Long transcripts that span the cluster length can potentially form dsRNA segments that may trigger piRNA processing.
